# Dataset on the reproducibility of UHPC mechanical properties under a fixed recipe with controlled production variability

**DOI:** 10.1038/s41597-026-07805-z

**Published:** 2026-07-15

**Authors:** Farzad Rezazadeh, Amin Abrishambaf, Gregor Zimmermann, Andreas Kroll

**Affiliations:** 1 Department of Measurement and Control Engineering, University of Kassel, Kassel, Germany; 2 QuantumFusion GmbH, Kassel, Germany; 3 MAITERIA UG, Kassel, Germany

## Abstract

A dataset was collected to investigate the reproducibility of ultra-high performance concrete (UHPC) quality under controlled variability, since UHPC mechanical properties can vary even when using a fixed mix formulation in production. This dataset is the first open-access dataset in the UHPC community to take a holistic view of the UHPC production process and associated production variability, featuring a relatively large number of systematically designed experiments under a fixed recipe. A reference UHPC recipe, designed for a 28-day compressive strength of 120 MPa, was used to produce 150 experimental records, while the quality, storage conditions, and dosing accuracy of raw materials, along with mixing parameters and curing conditions, were systematically varied to emulate real-world production environments. For each batch, five fresh UHPC properties and the average mixer power were recorded, and compressive and flexural strengths at 24 hours and at 28 days were measured using standardized methods under controlled laboratory conditions. By linking process variability to mechanical outcomes under the fixed reference recipe, the dataset supports reproducibility analysis in UHPC production and provides reusable data for machine learning, uncertainty modeling, and digital-twin development.

## Background & Summary

Ultra-high performance concrete (UHPC) is an advanced cement-based composite characterized by high compressive and flexural strength, very low permeability, and enhanced durability relative to conventional concrete^1^. These attributes enable the creation of lighter, more slender structural elements with longer service lives^2^, making UHPC advantageous for applications such as bridge decks, facade panels, and structural rehabilitation. However, the dense microstructure and low water-to-binder ratio that impart UHPC’s performance also render it sensitive to production variations^3^. Achieving consistent mechanical properties is challenging because factors such as raw-material quality^4^, raw-material storage conditions, potential raw-material dosing errors, and mixing and curing conditions all influence the hardened material.

The high cost of raw materials and the high dimensionality of this parameter space, combined with the 28-day curing period and the cost associated with destructive testing, limit the number of data points that can be practically collected^5^. Existing datasets intended to investigate cementitious composites have typically been structured to isolate a subset of factors and to emphasize mix proportions^6–8^. Consequently, the characterization of interactions among raw-material storage conditions, potential raw-material dosing errors, and mixing and curing conditions has been limited. In addition, cross-source compilations^8–15^ have been produced by merging experiments conducted under heterogeneous protocols, thereby introducing potential redundancies and inconsistencies in material quality, storage conditions, and production methods (Table 1).

**Table 1:** Overview of representative UHPC/HPC datasets.

Study / Dataset	Data Source & Size	Key Variables (Inputs → Outputs)
Sun *et al*.^6^	Laboratory experiments; 9 UHPC mix designs	Time-series signals (autogenous & plastic shrinkage under sealed/unsealed conditions; chemical shrinkage; internal RH & temperature; TGA) + mix/curing descriptors → early-age shrinkage metrics; compressive strength; elastic modulus; Poisson’s ratio.
Yeh, 2007^7^	Laboratory experiments; 103 mixes	Mix ingredients (cement, slag, fly ash, water, superplasticizer, coarse & fine aggregate) → slump, flow, and 28-day compressive strength.
Yeh, 1998^8^	Literature-compiled HPC dataset; 1030 data points (17 studies)	8 variables (cement, blast-furnace slag, fly ash, water, superplasticizer, coarse & fine aggregate + curing age) → compressive strength.
Aydın *et al*.^9^	Literature-compiled UHPC dataset; 890 data points	13 inputs (cement, slag, fly ash, silica fume, limestone powder, quartz powder, nano-silica, aggregate, water, fiber, superplasticizer, temperature, age) → compressive strength.
Aylas-Paredes *et al*.^10^	Literature-compiled UHPC dataset; ~ 1300 mixes (from >55 studies)	Dozens of mix-composition variables (cement, multiple SCMs, fibers, etc.) → 28-day compressive strength.
Xu *et al*.^11^	Literature-compiled UHPC dataset; 219 data points (19 studies)	10 inputs (five component-to-cement ratios, three fiber volume fractions, curing time, specimen shape) → compressive strength.
Qian *et al*.^12^	Literature-compiled UHPC dataset; 317 data points	21 inputs covering cement properties, fine- and coarse-aggregate contents, supplementary cementitious materials, water, superplasticizer, maximum aggregate size, polystyrene- and steel-fiber contents and geometries, and curing time → flexural strength.
Mahjoubi *et al*.^13^	Literature-compiled UHPC dataset; 1228 records	24 inputs in 4 groups: cementitious system, fiber variables, curing time, specimen dimensions → 28-day compressive strength; 28-day flexural strength; mini-slump flow; 28-day porosity.
Golafshani *et al*.^14^	Literature-compiled recycled aggregate concrete dataset; 2644 data points	Extensive mix-design features (cement content, *w*/*b*, recycled vs. natural aggregate ratios, SCM contents, admixture dosage; typical 28-day age) → compressive strength.
Kashem *et al*.^15^	Literature-compiled UHPC dataset (Mendeley Data); 810 mixtures	13 inputs (cement, age, water, nano-silica, quartz powder, limestone powder, aggregate, slag, superplasticizer, fiber, temperature, fly ash, silica fume) → compressive strength.
Rezazadeh *et al*.^28^	Original experimental UHPC dataset; 150 experimental records	23 variables per batch (fixed proportions; 5 fresh UHPC properties; mixer energy; curing regime) → compressive & flexural strengths at 24 hours and at 28 days.

Each row reports the dataset, data source and size, and the principal variable groups (inputs  → outputs). Literature-compiled datasets primarily emphasize mix-proportion diversity, whereas single-laboratory datasets isolate specific factors. The created dataset in this study (Rezazadeh *et al*.^28^) is distinguished by an invariant UHPC recipe with multifactorial production variability to enable reproducibility analysis.

To address the challenge of reproducibility of UHPC with designed mechanical properties considering all potential influencing factors, a dataset was collected that records every step from raw materials to cured material properties, all under a fixed UHPC recipe designed for facade panels with a target compressive strength of 120 MPa. In total, 150 experimental records of UHPC were systematically produced under this fixed recipe while deliberately varying raw-material quality, storage conditions, dosing accuracy, and curing procedures to capture real-world production uncertainties. For each batch, the average mixer power consumption and fresh UHPC properties (temperature, electrical conductivity, air content, slump-flow diameter, and funnel run time) were measured, along with mechanical properties at two ages: compressive and flexural strength at 24 hours, and compressive and flexural strength after 28 days of curing. Compiled from many batches under different process conditions, the dataset spans the high-dimensional parameter space encountered in UHPC production, offering insights into why a fixed recipe can sometimes fail to meet its design targets despite identical nominal proportions.

The experimental campaign underlying this dataset has already supported several methodological studies, but those studies used selected batch sets and input spaces for specific modeling or feature-selection objectives. Using the initial 50-batch Taguchi-screening phase, with 44 batches retained for model comparison after excluding batches with incomplete fresh-UHPC measurements, Rezazadeh *et al*.^16^ built a modeling pipeline integrating mix design, fresh UHPC properties, and curing conditions, and demonstrated using gradient boosting with recursive feature elimination that process parameters such as curing conditions and mixer power consumption are more influential predictors of 28-day compressive strength than the recipe proportions. Using the same 50-batch screening phase and a 16-factor candidate feature set, the same authors introduced an Ensemble-based Feature Importance Determination (E-FID) framework^17^, which aggregates multiple feature-selection methods and consistently identified curing conditions (specifically the temperature and curing regimen from day 2 to 28) as the most critical factors influencing final strength. Using the extended campaign after preprocessing, correlation-based input reduction, and outlier exclusion, i.e., a 139-record set with 16 candidate input variables, they also addressed the challenge of high-dimensional, small-sample modeling by developing a search-space-constrained variant of the NSGA-II algorithm^18^. By incorporating domain knowledge from the E-FID framework into its initialization, this approach improved prediction accuracy and stability for both compressive and flexural strength models. A further monitoring study^19^ used the same 139-record set derived from the same 150-batch campaign and an 11-variable process-input space after excluding anomalous or incomplete batches and removing highly correlated sand and filler variables; the study benchmarked machine-learning models, interpreted process sensitivities using SHAP/ALE, and evaluated a batch-specific curing-recommendation system.

The present data descriptor has broader scope than those prior analyses because it publishes the complete 150-batch experimental record together with the metadata and provenance of its creation, including the material system and reference mix, three-phase DoE, data-acquisition and testing protocols, raw unscaled measurements, variable definitions and units, missing-value and outlier annotations, and reusable DoE/post-processing code. Because the dataset documents systematic variations in raw-material quality, storage conditions, dosing errors, and curing conditions, it primarily supports investigations into why a fixed UHPC recipe sometimes fails to achieve its intended mechanical performance. Other potential reuse cases include benchmarking single and multi-objective feature selection algorithms and machine learning models in high-dimensional, limited-sample scenarios; exploring transfer learning to other UHPC recipes; studying how variations in raw-material storage, dosing errors, and curing protocols influence fresh UHPC properties and mixing energy; and developing digital twins for predictive quality control in cementitious materials.

## Methods

### Material Characteristics and Mix Composition

The reference UHPC mixture employed in this study was developed for the production of facade panels with a target compressive strength of 120 MPa at day 28 of the curing process. The material system was selected to represent established UHPC matrix-design practice for precast and facade-panel applications. Such mixtures commonly rely on a high Portland-cement content, silica fume as a reactive microfiller for matrix densification, fine quartz fillers and graded silica sands for particle-packing optimization, a low water content relative to the powder-rich matrix, and a polycarboxylate-based superplasticizer to secure particle dispersion and flowability^4^. UHPC standards and recommendations generally define performance requirements, material qualification, and test procedures, while the exact mixture composition remains application-specific. The selected constituents therefore provide an industrially relevant UHPC reference matrix: white 42.5R Portland cement is appropriate for architectural facade elements requiring high early strength and controlled surface appearance, whereas the high-purity silica fume, quartz fillers, and silica sands reflect commercially used raw-material classes for dense UHPC matrices. The selected material-quality and dosage ranges were chosen to represent practical supply, storage, and handling variations while preserving one fixed reference mix for reproducibility analysis.

**Fig. 1:**
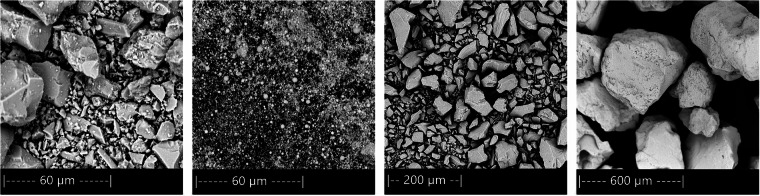
Scanning electron micrographs of the constituents used in the reference UHPC mixture are shown. From left, the micrographs show (i): white Portland cement (Type I, 42.5R), (ii) silica fume (>95.5% SiO_2_; silica for matrix densification), (iii) quartz filler (filler type I; 99.6% SiO_2_; *d*_50_ = 27 *μ*m), and (iv) quartz sand (*d*_50_ = 0.32 mm; 99.2% SiO_2_). The particle-scale morphology is presented as context for packing-density and porosity considerations in the reference mix.

It was composed of white Portland cement (600 kg/ m^3^), silica fume (100 kg/ m^3^), quartz fillers (450 kg/ m^3^ combined), two silica-based sands (1,100 kg/ m^3^ combined), water (195 kg/ m^3^), and a polycarboxylate-based superplasticizer (21.5 kg/ m^3^), from which (scaled) a batch volume of 15 L of UHPC product was obtained. The cement was a 42.5R white Portland type I. Silica fume with >95.5% SiO_2_ content was used to densify the matrix and reduce porosity, thereby supporting mechanical performance and durability. Quartz powder with 99.6% SiO_2_ and a median particle size *d*_50_ = 27 *μ*m was used as filler type I. Filler type II was included to further promote workability and enhance strength. Two grades of silica-based sand, with *d*_50_ values of 0.32 mm and 0.94 mm and a purity of 99.2% SiO_2_, were incorporated; the graded blend was selected to optimize particle packing and minimize voids, thereby yielding a dense UHPC microstructure. Representative scanning electron microscopy (SEM) images of the raw materials are shown in Fig. 1.

A polycarboxylate-based superplasticizer was incorporated to secure the required flowability at a low water-to-binder ratio. In this manner, fresh UHPC workability was ensured and final mechanical performance was supported. Collectively, these constituents were combined, and a UHPC mixture suitable for advanced construction applications – where high strength, durability, and workability are required – was obtained.

### Simulating Variability in UHPC Production with the Reference Mix Design

Parameters ranging from UHPC raw materials and potential measurement errors in raw-material dosing to the mixing and curing conditions (Table 2) were investigated to evaluate their effects on compressive and flexural strengths. The cement and silica fume contents were kept constant, whereas the remaining factors were varied to emulate real-world conditions and possible material inconsistencies. Cement reactivity (CR) was represented by either a new delivery (Level 2) or a delivery stored for six months (Level 1).

**Table 2:** Selected input factors and their levels used in the UHPC experiments are summarized.

Group	Factor	Symb.	Unit	Level 1	Level 2	Level 3	Level 4	Level 5
**Material quality**	Cement reactivity	CR	Class	1	2	—	—	—
	Graphite	GRP	kg	0.045	0.000	0.090	0.135	0.225
	Ingredient moisture	IM	%	+4	(2.925 kg)	+8	+12	+15
	Ingredient/Water temperature	IT	^°^C	10	20	25	30	40
**Particle size distributions** & **measurement errors**	Sand type I	SAI	%	+15	(6.000 kg)	−10	+5	−15
	Sand type II	SAII	%	−15	(10.500 kg)	+10	−5	+15
	Filler type I	FLI	%	−15	(6.000 kg)	+10	−5	+15
	Filler type II	FLII	%	+15	(0.750 kg)	−10	+5	−15
	Superplasticizer	SPP	%	−10	(0.323 kg)	−5	+10	+5
**Mixing conditions**	Mixing speed	MS	rpm	200	350	500	350	350
	Mixing duration	MD	s	300	300	300	210	480
	Eng_1_: Mixing regime	MR	Class	1	2	3	4	5
**Curing conditions, first 24 h**	Curing temperature, day 1	CT_1_	^°^C	20	20	10	30	40
	Curing class, day 1	CC_1_	Class	S-RH	S-PW	S-PW	S-PW	S-PW
	Eng_2_: Curing condition, day 1	CD_1_	Class	1	2	3	4	5
**Curing conditions, days 2–28**	Curing temperature, days 2–28	CT_28_	^°^C	20	20	10	30	40
	Curing class, days 2–28	CC_28_	Class	S-PW	S-W	S-W	S-W	S-W
	Eng_3_: Curing condition, days 2–28	CD_28_	Class	1	2	3	4	5

Level 2 corresponds to the reference mix, while the others represent variations around this reference to cover a broad range of material, environmental, and processing conditions. Six original process variables, MS, MD, CT_1_, CC_1_, CT_28_, and CC_28_, were consolidated into three categorical engineered descriptors, MR, CD_1_, and CD_28_, reducing the DoE factor space from 15 to 12 while retaining the component settings in the table. (Eng_*i*_: *i*-th engineered factor; S-RH: specimens at 90% relative humidity; S-PW: specimens encased in plastic wrap; S-W: specimens submerged in water).

Variations in silica-fume quality were represented by adding graphite (GRP) as a carbonaceous surrogate. The GRP levels in Table 2 are batch masses for the 15 L mixtures; the maximum dosage of 0.225 kg corresponds to 15 kg/ m^3^, i.e., 15 wt.% relative to the silica-fume content of 1.50 kg per batch (100 kg/ m^3^). This upper bound was selected from the common qualification criterion that silica fume for concrete applications should contain at least 85 wt.% SiO_2_^20,21^. The remaining 15 wt.% was therefore used as a conservative equivalent impurity fraction. Because the non-SiO_2_ fraction of commercial silica fume may include oxides, moisture, loss-on-ignition constituents, and other minor phases, the graphite addition denotes a controlled carbon-rich quality perturbation; the actual carbon content of the selected high-purity silica fume (>95.5% SiO_2_) is expected to be lower. The selected range therefore spans the reference high-purity condition and a conservative upper-bound scenario for investigating how carbonaceous contamination may influence fresh-UHPC response, mixing behavior, matrix densification, and mechanical performance.

The moisture condition of the sand, denoted as ingredient moisture (IM) in Table 2, was varied by using dry solid raw materials as the experimental reference state and adding a prescribed amount of water to the batch, corresponding to defined moisture levels from oven-dry to saturated surface-dry sand. This procedure was used to isolate the effect of sand moisture, which is particularly relevant for industrial UHPC production because aggregates may be stored outdoors or in humid environments and may therefore be exposed to rain, drying, or changing ambient humidity. The added water entered the mixer as part of the effective batch water and emulated the water contribution introduced by moist sand during batching. The nominal water content of the fixed reference formulation was 195 kg/m^3^, corresponding to 2.925 kg for the 15 L laboratory batch and to a nominal water-to-binder ratio of *w*/*b*_nom_ = 195/(600 + 100) = 0.279, when cement and silica fume are considered as binder. This nominal recipe water was kept constant as the reference condition. Increasing IM therefore increased the effective water content and, consequently, the effective water-to-binder ratio. In the dataset, the nominal water content is given by the fixed reference mix, whereas IM records the batch-specific water content associated with the sand-moisture factor. For the data-file values expressed in kg/15 L, the added moisture-equivalent water is obtained as ${W}_{{\rm{add}}}={\rm{IM}}-2.925\,{\rm{kg}}$, and the corresponding effective water-to-binder ratio is $w/{b}_{{\rm{eff}}}={\rm{IM}}/10.5$, where 10.5 kg is the cement-plus-silica-fume binder mass in a 15 L batch. Thus, the dry reference state separates the effect of sand moisture from simultaneous changes in cement, silica fume, filler, and aggregate properties, allowing the influence of uncontrolled storage moisture on fresh UHPC behavior and hardened mechanical properties to be evaluated systematically.

In addition, the temperature of the ingredients (IT) was adjusted from 10 ^°^C (cold storage) to 40 ^°^C (hot storage) to reflect seasonal storage variations. For this purpose, all raw materials were conditioned in a climate chamber under controlled conditions for at least 48 hours prior to mixing to ensure attainment of the target temperature. These manipulations were intended to reproduce influences associated with uncontrolled open-air storage of raw materials. Gradations and contents of the fillers and sands were adjusted by ±5% to ±15% to simulate variations in the particle size distribution due to inconsistent deliveries and potential dosing errors for the sands and fillers; corresponding effects on matrix packing density were anticipated. Finally, the superplasticizer dosage (SPP) was altered to assess the impact of dosing inaccuracies on UHPC workability and mechanical properties.

**Fig. 2:**
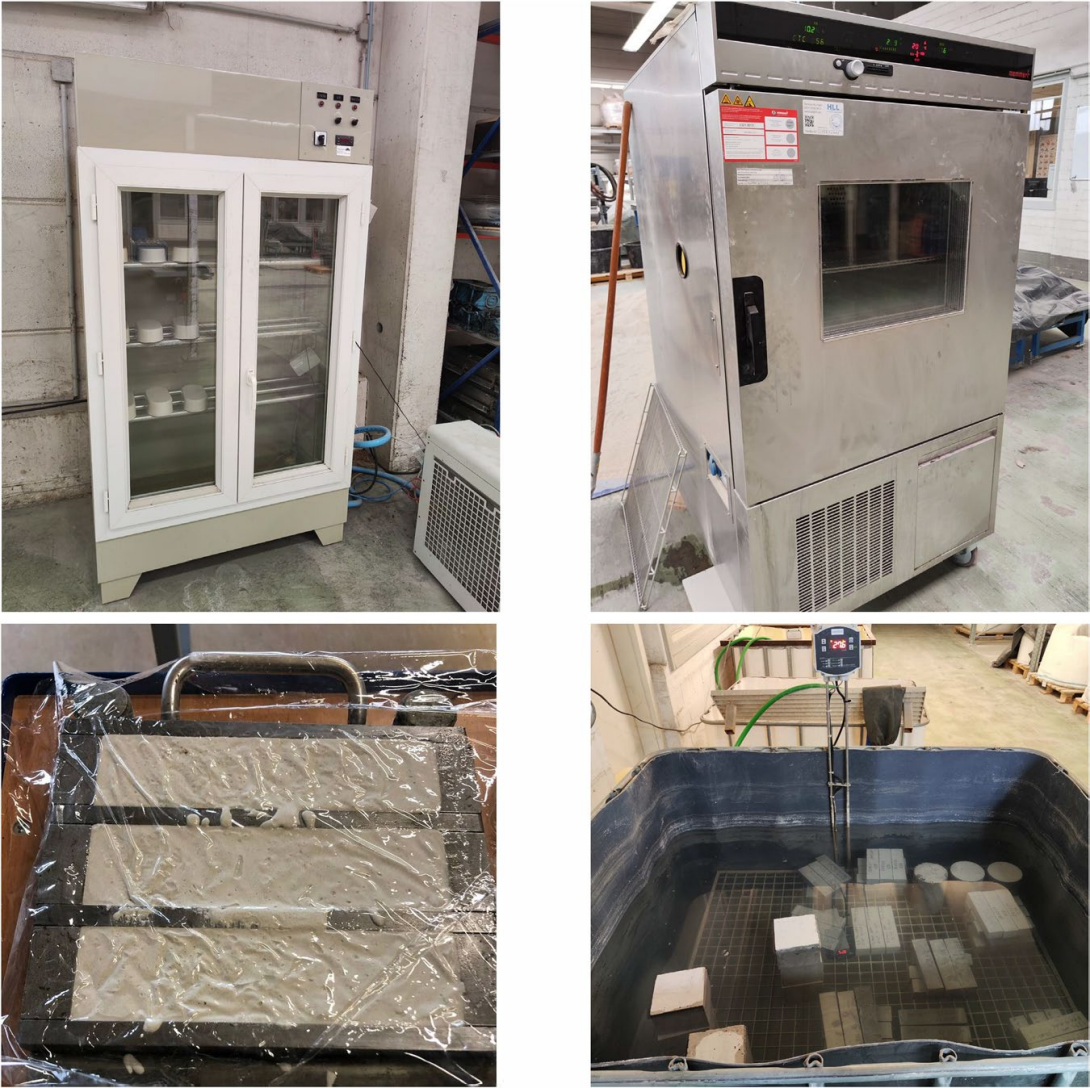
Environments used to acquire the curing variables recorded in the dataset: curing class (CC_1_, CC_28_) and curing temperature (CT_1_, CT_28_). Panels correspond to Day 1 conditions (top) and Days 2–28 conditions (bottom), matching the level definitions in Table 2, (top-left): Day 1 - specimens in a 90% RH cabinet (class S-RH); cabinet air temperature and RH were adjusted in the chamber, (top-right): Day 1 - molds sealed under plastic wrap (class S-PW) to suppress evaporation and stored at 10–40 ^°^C, (bottom-left): Days 2–28 - sealed specimens (class S-PW) stored in the experimental environment with a constant temperature of 20 ^°^C; chamber air temperature was logged at shelf height, (bottom-right): Days 2–28 - specimens submerged in a temperature-controlled water bath (class S-W); water temperature was read by the bath probe at mid-depth.

Mixing speed and mixing duration were varied to assess their roles in achieving the desired UHPC properties. Mixing intensities from 200 rpm to 500 rpm and durations from 210 s to 480 s were tested to emulate production variability and to promote a homogeneous mixture. To investigate the influence of curing conditions, specimens were subjected to distinct conditions and temperatures (Fig. 2). During the first 24 hours, fresh specimens were either maintained in a climate chamber at 20 ^°^C and 90% relative humidity or covered with a plastic sheet and stored at 10–40 ^°^C. After demolding, from day 2 to day 28, specimens were either wrapped in plastic at 20 ^°^C or submerged in water at 10–40 ^°^C. These curing variations were designed to emulate post-mixing environments that influence the final mechanical properties of UHPC. After aggregation of all potential inputs into a single feature pool, a total of 15 critical influencing factors were obtained (Table 2).

Owing to the sparsity of the input space, feature engineering was applied to process variables that act as coupled experimental states. Mixing speed (MS) and mixing duration (MD) were consolidated into the categorical mixing regime (MR) because, in an intensive mixer, the prescribed mixing protocol is governed by their combination. The speed-duration pair determines the imposed shear history, residence time, particle dispersion, agglomerate breakdown, paste homogenization, and frictional heat development during mixing. The five MR levels therefore represent the five executed operating regimes listed in Table 2, rather than an arbitrary numerical aggregation of two independent variables. Statistically, treating MS and MD as separate factors would imply a partially unobserved factorial speed-duration space, because only selected practical combinations were investigated. Encoding the executed combinations as MR reduces over-parameterization, avoids inference for untested speed-duration combinations, and preserves the experimental provenance because the original MS and MD settings remain explicitly reported in Table 2. The measured average mixer power (APW) was retained separately as a process-response variable, since it reflects the mixture-dependent resistance during mixing and is not equivalent to the prescribed speed-duration setting. The same rationale was used for curing: CT_1_ with CC_1_, and CT_28_ with CC_28_, were consolidated into CD_1_ and CD_28_, respectively, because curing temperature and exposure medium jointly define the thermal-moisture boundary condition at each curing stage. This reduced the DoE factor space from 15 to 12 variables while keeping the engineered factors physically interpretable and statistically tractable for the 150-batch campaign.

### Design of Experiments

**Fig. 3:**
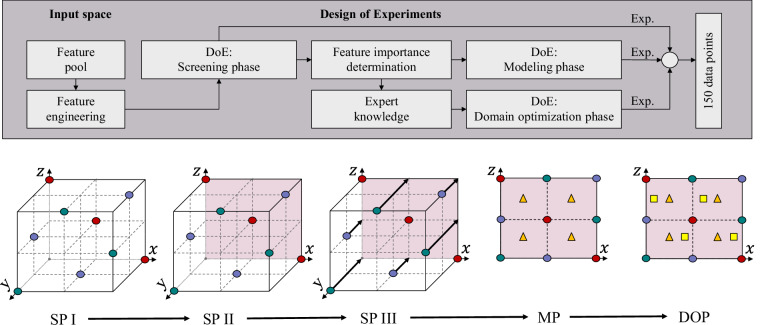
Three-phase design of experiments (DoE) workflow and its implementation for the ultra-high performance concrete data collection, (top): The three-phase DoE is shown to begin with input-feature pool creation and feature engineering and proceed through the screening, modeling, and domain-optimization phases, (bottom): A simplified illustration of the implemented three-phase DoE using L_9_ is provided. Here, L_9_ denotes the standard nine-run Taguchi orthogonal array (TOA) for three-level factors (often written L_9_(3^4^)); in this illustration it corresponds to nine experiments exploring three factors, each at three levels. In the screening phase (SP I), a TOA is used for factor screening. After experimentation, the least important factor (denoted *y*) and the most important factor (denoted *x*) are identified (SP II). Following removal of the least important factor, the existing TOA design is augmented with Latin hypercube sampling (LHS) in the modeling phase (MP) to achieve space-filling properties. Finally, in the domain-optimization phase (DOP), the most important variable, *x*, is positioned at different levels based on expert knowledge derived from SP.

Given the limited number of feasible experimental runs, the experimental design was constructed to maximize information capture and to cover the input space effectively so as to mitigate overfitting. To this end, a three-phase design of experiments (DoE) framework was established, comprising the screening, modeling, and domain-optimization phases (Fig. 3). In total, 150 batch experiments were performed across these three phases.

The three-phase structure was chosen to stage information gain across the campaign: in the screening phase, main effects were identified with a minimum number of experiments so that dimensionality could be reduced and resources were concentrated on the relevant factors; the foundational design space was provided by the screening phase and, therefore, in the modeling phase, the objective was augmentation of the designed input space from the first phase, with a focus on factor interactions for predictive modeling; in the domain-optimization phase, refinement was performed by targeting the most influential factors to close residual gaps in the explored design-input subspace and to render the overall coverage more uniform.

#### Screening phase

The principal objective of the screening phase was the identification of both negligible and influential factors. Given the constraint of 50 experimental runs in this phase, early-stage dimensionality reduction via removal of non-informative features was prioritized. Among the component settings retained in Table 2, cement reactivity (CR), curing class day 1 (CC_1_), and curing class days 2–28 (CC_28_) had two levels, whereas the remaining features had five levels each (Table 2). The L_50_ Taguchi orthogonal array (TOA)^22^ was employed to design the 50 experiments, as TOA methods are well established for screening with high-dimensional, small-sample settings and for identifying main effects without reliance on specific model assumptions^23^. Balanced level distributions were ensured by the TOA, an important property given the absence of prior information about factor significance.

Analysis of the screening data^17^ indicated that, within the factor ranges considered in the initial Taguchi L_50_ screening phase, the variations in cement reactivity (CR), mixing speed (MS), and mixing duration (MD) had negligible influence on the final mechanical properties of the reference UHPC, especially when compared with the curing-related variables. In the underlying feature-importance analysis^17^, curing conditions, especially the temperature and curing regime from day 2 to day 28, were consistently identified as the dominant factors, whereas CR, MS, and MD ranked among the less influential variables for both compressive and flexural strength. Moreover, the average mixer power consumption was retained in the dataset as an integrated descriptor of the mixing process and fresh-mixture resistance; this variable was more informative than the prescribed mixer speed and duration in the screening analysis^17^.

Consequently, these three factors were fixed in the modeling and domain-optimization phases to concentrate the limited experimental budget on the variables with the highest expected information gain. For the remaining experimental runs, raw materials were used directly after delivery, i.e., without six-month cement storage, and the mixing speed and mixing duration were set to the reference Level 2 conditions listed in Table 2. This decision improved the statistical efficiency of the subsequent design by reducing the dimensionality of the input space and allowing denser exploration of raw-material dosing, storage-temperature, fresh-property, mixer-power, and curing-condition effects.

This exclusion also defines a limitation of the dataset. The full 150-record dataset should not be interpreted as providing a complete independent assessment of cement-reactivity effects or nominal mixing-speed and mixing-duration effects beyond the screened levels. Possible interaction effects involving these factors may be under-represented because they were not varied in the later phases. Therefore, the conclusion is restricted to the present fixed UHPC recipe, cement type, cement-storage condition, EIRICH intensive mixer, and tested mixing window.

#### Modeling phase

To achieve a more uniform coverage of the input space relative to the existing TOA design from the screening phase, the experimental plan was augmented by adding 50 new experiments to the Taguchi L_50_ design. This augmentation combined the structured orthogonality of the TOA with the space-filling characteristics of Latin hypercube sampling (LHS)^24^, thereby enabling systematic exploration for modeling UHPC properties (Algorithm 1).

##### Algorithm 1

Augmentation of an experimental design while preserving Latin hypercube sampling assumptions^25,26^.

The objective was to add *N*_II_ new points to the existing design matrix ${{\bf{X}}}_{{\rm{existing}}}\in {{\mathbb{R}}}^{{N}_{{\rm{I}}}\times K}$ from the screening phase, where *N*_I_ = 50 denotes the number of screening-phase experiments and *K* the number of variables, yielding an augmented design of *N*_total_ = *N*_I_ + *N*_II_ samples. The original Taguchi design, with discrete levels in {1, 2, 3, 4, 5}, was first normalized to [0, 1] to ensure compatibility with LHS. The normalized design was then partitioned into *N*_total_ equal intervals (strata) for each variable,

1 $$S=\left\{\left[\frac{s-1}{{N}_{{\rm{total}}}},\,\frac{s}{{N}_{{\rm{total}}}}\right)\,| \,s=1,\ldots ,{N}_{{\rm{total}}}\right\}.$$

with half-open intervals employed to avoid overlap and to maintain clear stratification. Existing points were mapped to their corresponding intervals, for each variable *j*, to determine the occupied sets *S*_occupied,*j*_; the unused intervals were then identified as *S*_unused,*j*_ ← *S*⧹*S*_occupied,*j*_.

Candidates were generated only from strata (intervals) that were unused by the existing Taguchi design. For each variable *j*, one value was drawn uniformly from every *s* ∈ *S*_unused,*j*_ to create a list *B*_*j*_, and each list *B*_*j*_ was randomly permuted. By aligning the permuted lists across variables by index, candidate *K*-tuples were produced in which every variable occupies a distinct, previously unused stratum. This pass was repeated *R* times, and all candidates were pooled as ${\mathscr{P}}$. For each ${\bf{p}}\in {\mathscr{P}}$, the minimum Euclidean distance to the existing points, ${d}_{\min }({\bf{p}})$ in Eq. (3), was computed and ${\mathscr{P}}$ was sorted in descending order of this score. Candidates were accepted sequentially into **X**_new_ provided that their strata were still unused in every dimension (the sets *S*_selected,*j*_). In this manner, LHS-like stratification was enforced for the newly added points while the strata already occupied by the existing Taguchi design were respected.

Uniform coverage of the input space was promoted using the S-optimality criterion^25,26^, which maximizes the minimum pairwise Euclidean distance among all design points:

2 $$\max \mathop{\min }\limits_{\begin{array}{c}1\le i < j\le {N}_{{\rm{I}}}+{N}_{{\rm{II}}}\end{array}}{\left\Vert {{\bf{p}}}_{i}-{{\bf{p}}}_{j}\right\Vert }_{2},$$

where **p**_*i*_ and **p**_*j*_ denote points in the augmented design. For each candidate point ${{\bf{p}}}_{l}\in {\mathscr{P}}$, the minimum Euclidean distance to the existing points was computed as

3 $${d}_{\min ,l}\,=\,\mathop{\min }\limits_{i=1,\ldots ,{N}_{{\rm{I}}}}{\parallel {{\bf{p}}}_{l}-{{\bf{X}}}_{{\rm{existing}}}[i,:]\parallel }_{2}.$$

Candidates were then sorted in descending order of *d*_min,*l*_ and selected sequentially to form **X**_new_ (the set of *N*_*II*_ newly added points). To preserve the LHS property, *S*_selected,*j*_, the set of intervals already occupied by existing points or previously selected new points in that dimension, was maintained for each variable *j* and initialized as *S*_selected,*j*_ := *S*_occupied,*j*_. A candidate **p**_*l*_ was accepted only if interval (*p*_*l*,*j*_) ∉ *S*_selected,*j*_ for all *j*.

The augmented design was obtained by combining the existing and newly selected points, **X**_augmented_ ← **X**_existing_ ∪ **X**_new_, after which the normalized values were rescaled to the original discrete levels and rounded to the nearest integer, as shown in Table 2.

The computational cost of the DoE augmentation was also recorded for the implemented case. Augmenting the initial Taguchi design with *N*_I_ = 50 experiments by *N*_II_ = 50 additional LHS-based design points required approximately 200 s wall-clock time on a workstation equipped with an Intel(R) Core(TM) i9-10900X CPU at 3.70 GHz and 64 GB RAM. This runtime includes candidate generation from unused LHS strata, computation of the distance-based ranking criterion, sorting of the candidate pool, and sequential acceptance of the final design points.

#### Domain-optimization phase

To further examine the influence of curing conditions – identified during screening as the most impactful set of factors^17^ – the domain-optimization phase was designed. In parallel with the modeling phase, each of the 50 experiments was replicated under alternative curing conditions determined by expert knowledge. Thus, in each of these 50 trials (the modeling-phase experiments), two specimen sets were prepared:

- Original curing conditions: those specified in the modeling-phase design.
- Alternative curing conditions: new conditions based on expert insights not previously explored in the screening or modeling phases.

By curing each mixture under two distinct sets of conditions, two sets of outputs were generated from each experimental run in the modeling phase. The three-phase approach (screening, modeling, and domain-optimization) thereby yielded a total of 150 data points. All experiments were conducted in the laboratory under controlled conditions, using the same mixer, the same mixing tool, and the same personnel. The environment was maintained at a constant temperature of 20 ^°^C throughout the experimental process to minimize external variability and ensure that observed differences in the UHPC properties were primarily due to controlled experimental factors.

### Data acquisition

The DIN EN standards cited in this section are used as method references to define reproducible sampling, apparatus geometry, specimen preparation, curing, measurement timing, and loading conditions. DIN EN denotes the German implementation of a European Standard (EN); in the present dataset, the relevance of these references is the standardized test procedure rather than a national performance classification. The DIN EN 12350 series specifies the fresh-concrete test methods used here, including sampling/common apparatus, slump-flow, V-funnel efflux time, and pressure-method air-content measurement. DIN EN 12390-2 defines specimen making and curing procedures for hardened-concrete strength testing, whereas DIN EN 196-1 defines the prism geometry and flexural/compressive loading configuration used for the mechanical measurements. These standards therefore support within-study reproducibility and cross-laboratory comparability of the measured variables, while the UHPC factor levels and performance targets remain defined by the present experimental design.

#### Mixing conditions

**Fig. 4:**
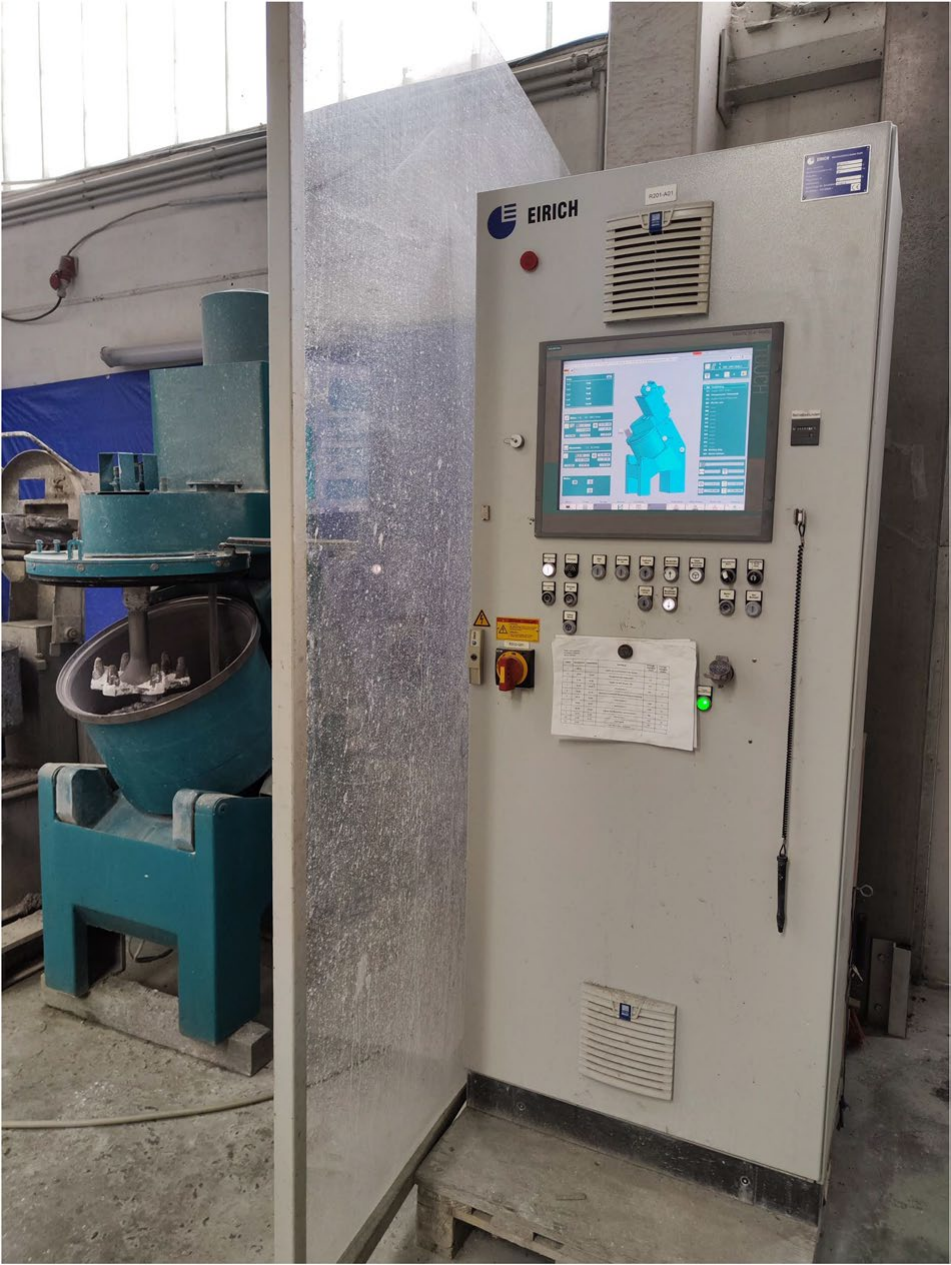
PLC-controlled EIRICH intensive counter-current pan mixer used for all batches. Tool speed and mixing duration were manually set; instantaneous motor power *P*(*t*) was logged at 1 Hz by the controller and integrated to obtain the total mixing energy *E* (PLC: Programmable logic controller).

Mixing was carried out in an EIRICH intensive counter-current pan mixer (Fig. 4). The mixer’s speed and mixing duration were manually set. The controller logged the instantaneous motor power *P*(*t*) with 1 Hz sampling. For each batch, the total mixing energy was computed as

4 $$E={\int }_{0}^{T}P(t)\,{\rm{d}}t\approx {\sum }_{i=1}^{N}{P}_{i}\,\Delta t.$$

#### Fresh UHPC properties

**Fig. 5:**
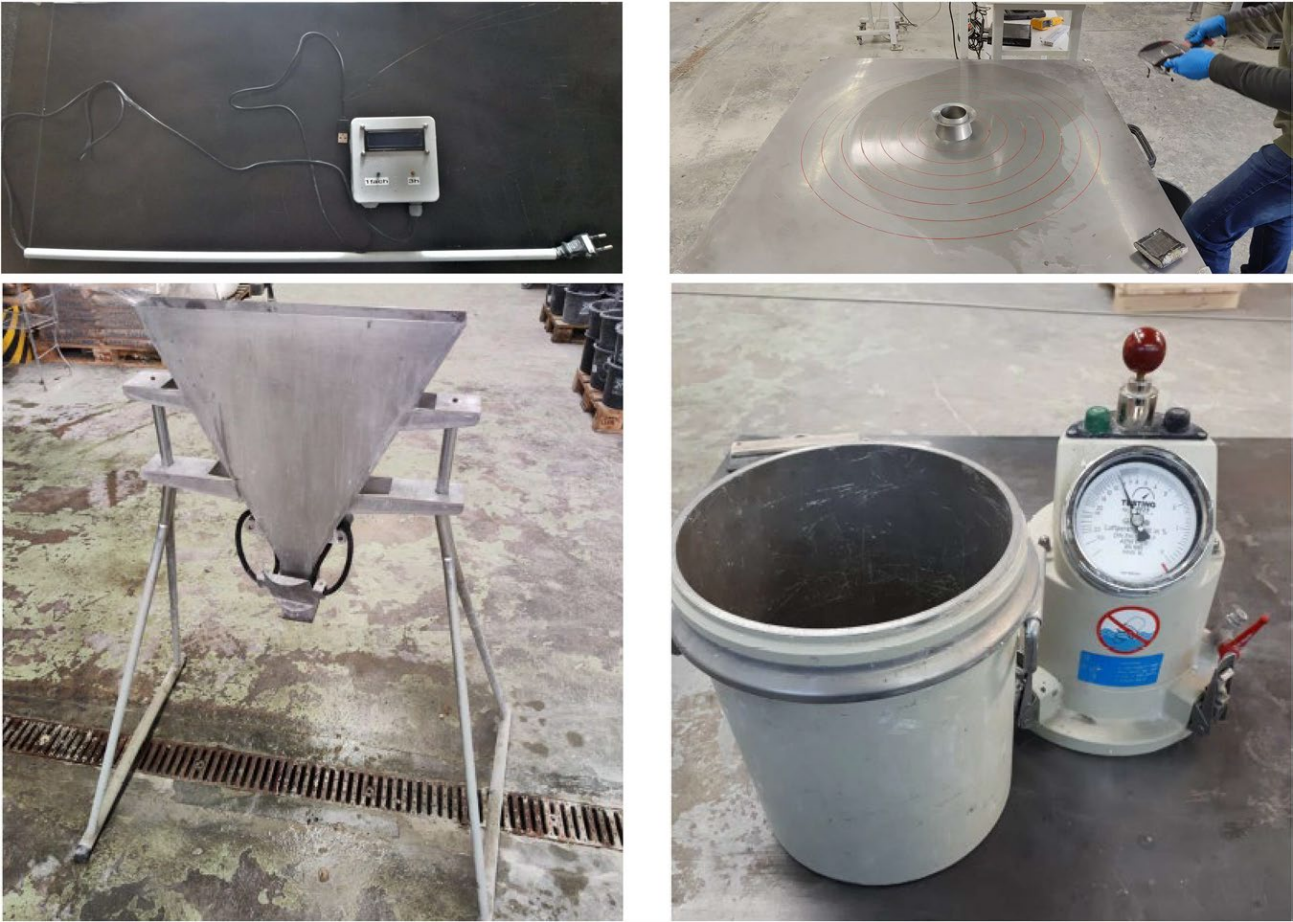
Equipment used to acquire fresh UHPC properties of the UHPC batches, (top-left): Low-cost prototypical electrical-conductivity device comprising a two-electrode immersion probe and handheld logger; three readings were taken at predefined positions and averaged, (top-right): DIN EN 12350-8:2019 slump-flow setup with mini cone on a non-absorbent, marked plate; two orthogonal diameters and the *T*_200_ time were recorded, (bottom-left): DIN EN 12350-9:2010 efflux time was measured from trapdoor opening until discharge was complete, (bottom-right): DIN EN 12350-7:2022-05 type B pressure air meter (8 L bowl, hand pump, analog dial) used to determine fresh UHPC air content.

Immediately after mixing, five fresh UHPC properties were measured (Fig. 5). The batch was first discharged into a rigid, non-absorbent container that served as the sampling reservoir for the tests.

*Fresh UHPC temperature*. Fresh UHPC temperature was measured in accordance with DIN EN 12350-1:2019 (*Testing fresh concrete – Part 1: Sampling and common apparatus*), using a partial-immersion liquid-in-glass thermometer. The sensing bulb was embedded ~50–75 mm below the surface; the reading was taken after the thermometer indication had settled. The instrument accuracy of ±0.5 ^°^C exceeded the EN requirement of ±1 ^°^C.

*Electrical-conductivity response*. The electrical-conductivity response of fresh UHPC was measured with a low-cost prototypical device (Fig. 5) at three fixed positions inside the container. The batch value reported was the arithmetic mean of the three readings. The electrodes were supplied with a 0–5 V square-wave PWM signal at approximately 1 kHz, and the divider node was sampled by a 12-bit analog-to-digital converter referenced to 5 V. The public data file reports the averaged ADC response *X*; the corresponding device voltage *v* is obtained as

$$v=\frac{X}{2048}\cdot 5\,{\rm{V}}.$$

Thus, EC represents an ADC-derived device response of the fresh mixture. The circuit concept (AC-PWM excitation and open-source, low-cost measurement logic) followed Visco *et al*.^27^; see their device-circuit description and full schematics for implementation details. The device was used without calibration to absolute conductivity units because the present study used EC as a relative process indicator across batches produced with the same UHPC recipe and measured with one unchanged device. Reproducibility within the present campaign is supported by the fixed probe and electrode geometry, excitation signal, ADC reference, measurement positions, and averaging protocol. Direct numerical comparison with conductivity values from other studies requires either the same device and measurement protocol or a device-specific calibration curve against reference solutions or an independently calibrated conductivity meter, with temperature correction and electrode/sample geometry documented. In the absence of such calibration, cross-study use of EC should be restricted to normalized trends or within-study rankings of fresh-mixture electrical response.

*Slump-flow test*. Slump-flow was determined using a mini-cone procedure adapted from DIN EN 12350-8:2019 (*Testing fresh concrete – Self-compacting concrete – Slump-flow test*). The slump-flow diameter was reported as the mean of two orthogonal spreads; the *T*_200_ time (time to reach a 200 mm spread) was recorded with a stopwatch (Fig. 5).

*V-funnel (efflux) time*. Efflux time was measured using a manual V-funnel in accordance with DIN EN 12350-9:2010 (*Testing fresh concrete – Self-compacting concrete – V-funnel test*). The funnel was dampened and closed, then filled and struck off flush; after 10 ± 2 s, the trapdoor was opened and the flow time was measured from opening until discharge was complete (Fig. 5).

*Air content*. Air content was measured in accordance with DIN EN 12350-7:2022-05 (*Testing fresh concrete – Air content – Pressure methods*) using a manual pressure air meter (type B style; 8 L bowl). The bowl was filled and consolidated as specified in the standard (Fig. 5).

#### Curing conditions

Specimen making and standard curing procedures are described in DIN EN 12390-2:2019 (*Testing hardened concrete – Making and curing specimens for strength tests*). For each batch, two descriptors were recorded to capture the curing conditions. The curing class (CC_1_ for Day 1 and CC_28_ for Days 2–28) denoted the type of curing environment. The curing temperature (CT_1_, CT_28_) was recorded in degrees Celsius for the corresponding period. Each such (CC, CT) pair was also encoded as a single categorical variable (CD_1_ for Day 1 and CD_28_ for Days 2–28) according to Table 2.

For Day 1 curing (first 24 h), the S-RH class was maintained at 20^°^C in a 90.0% relative-humidity cabinet, whereas the S-PW class used sealed specimens stored in a temperature-controlled cabinet set to a target temperature between 10^°^C and 40^°^C, depending on the assigned level. For Days 2–28, sealed curing (S-PW) was carried out at an ambient room temperature fixed at 20 ^°^C, while water curing (S-W) was conducted with specimens submerged in water baths maintained at temperatures between 10^°^C and 40^°^C according to the specified level (Fig. 2).

#### Mechanical properties

**Fig. 6:**
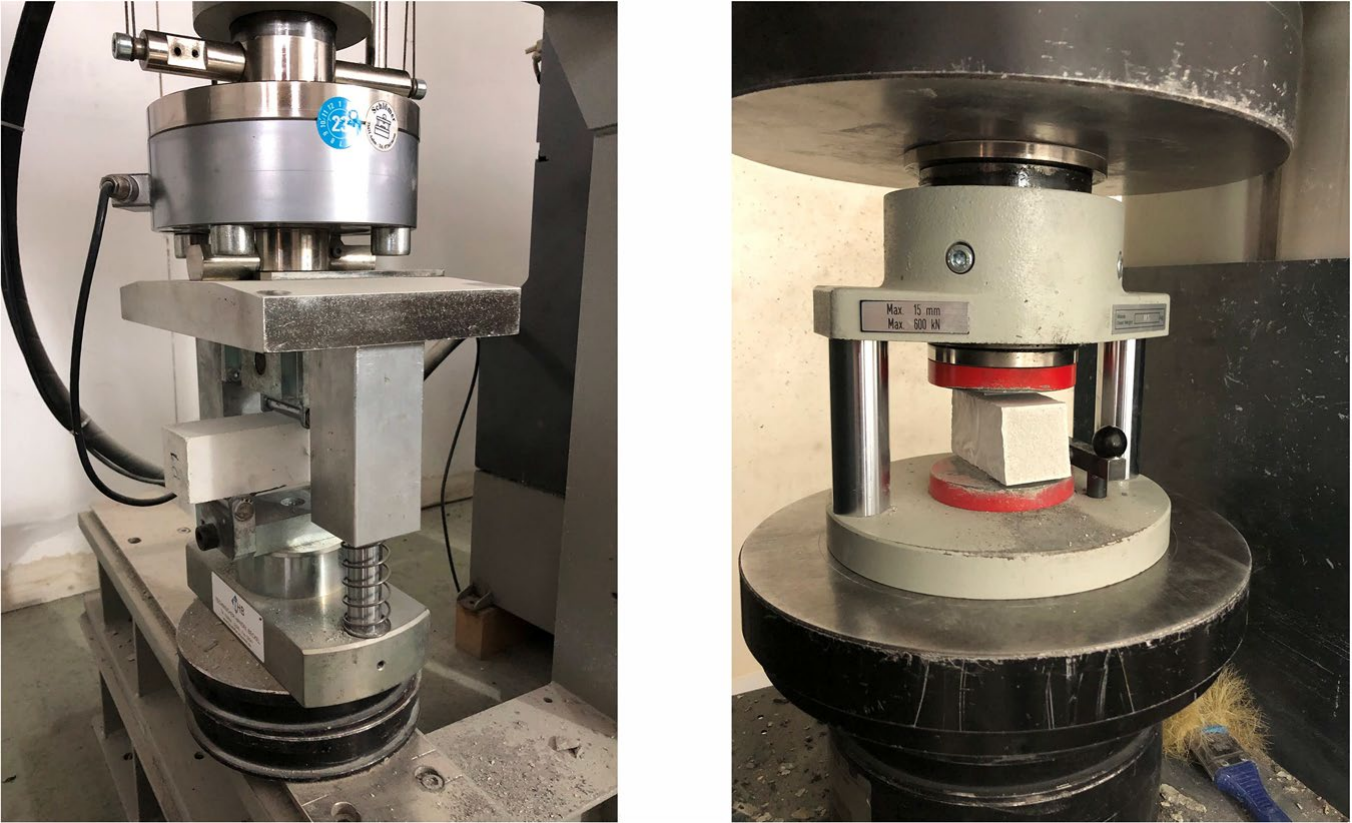
Mechanical-test setup used for 24-hour and 28-day properties, (left): three-point bending setup for 40 × 40 × 160 mm prisms (support span of 100 mm) used to determine the flexural strength at 24 h and 28 d, (right): compression test on 40 × 40 × 80 mm halves between plane steel platens (spherically seated upper platen) to determine compressive strength at 24 h and 28 d. Reported strengths are the means of three prisms (flexural) and six halves (compressive).

In accordance with DIN EN 196-1:2016-11, fresh UHPC from each experiment was cast into 40 × 40 × 160 mm prisms (two sets of three specimens per batch, one set for 24-hour and one set for 28-day mechanical properties measurement) and cured under the specified regime. At 24 h and again on day 28 of the curing process, flexural strength was determined first, followed by compressive strength on the two halves of each prism (Fig. 6).

Flexural strength tests were performed in three-point bending with a 100 mm support span at a loading rate of 50 ± 10 N s^−1^ (Fig. 6-left). The flexural strength was computed as

5 $${f}_{{\rm{flex}}}=\frac{3F\,L}{2\,b\,{h}^{2}},$$

with *F* the maximum load (N), *L* = 100 mm, and *b* = *h* = 40 mm. After fracture, each prism was halved and the compressive strength of each 40 × 40 × 80 mm half was measured between plane steel platens (spherically seated upper platen) at 2400 ± 200 N s^−1^ (Fig. 6-right). Compressive strength was computed as

6 $${f}_{{\rm{c}}}=\frac{F}{A},\,\,\,\,A=40\times 40=1600\,{{\rm{mm}}}^{2}.$$

Flexural strengths are reported as the mean of three prisms of the same batch; compressive strengths are reported as the mean of six half-prisms.

## Data Records

The dataset is publicly available through the University of Kassel’s research data repository (DaKS) under the title *Mechanical Properties of Ultra-High Performance Concrete (UHPC)* (10.48662/daks-56.2, see also^28^). It comprises the following files:

- Ultra_High_Performance_Concrete_UHPC.xlsx / .csv – A dataset containing the raw (unscaled) measurements from 150 UHPC batch experiments. Each row represents one experimental record, and the file has columns corresponding to specific input factors or measured outcomes (covering all mix parameters, fresh UHPC test results, and strength measurements).
- README.txt – A plain-text metadata file that provides detailed context for the dataset, including the experimental background, definitions of each column/variable, and descriptions of the methodologies used for data collection.
- Design_of_Experiments_Code.zip – The archive contains a README for the DoE framework code, an R script implementing the Latin hypercube augmentation of the Taguchi screening design, and two Jupyter notebooks for post-processing and reproducing the analyses.

Within the dataset, any unmeasured value is denoted by a blank cell / empty field (no placeholder values are used). In addition, 11 of the 150 batch entries have been flagged by the data creators as anomalous records; these cases are annotated within the dataset to highlight their anomalous nature, serving as a notice to users regarding data quality.

## Technical Validation

The technical quality of the dataset was assessed at three complementary levels: (i) balance and coverage of the experimental design, (ii) adherence to standardized and well-characterized measurement procedures, and (iii) systematic screening for anomalous records, transcription errors, and implausible observations, together with an explicit characterization of the empirically observed ranges of all variables.

**Fig. 7:**
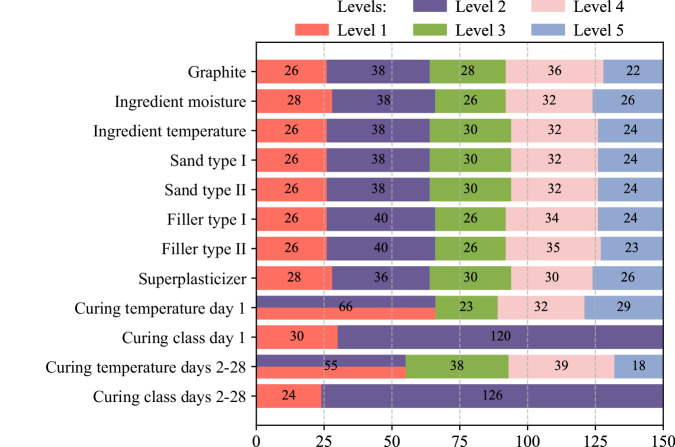
Distribution of factor levels in the collected dataset. Each bar shows the frequency across all 150 experiments for the corresponding factor, i.e., the bar heights sum to 150 per factor. CC_1_ and CC_28_ each have two levels. For CT_1_ and CT_28_, Levels 1 and 2 take the same numeric value (both 20 ^°^C).

The three-phase design of experiments (DoE) – comprising Taguchi-based screening, space-filling augmentation, and domain optimization – was constructed to promote orthogonality, space filling, and domain-informed refinement; in this section, balance is evaluated as an observed quality attribute of the completed 150-batch record. This validation step is relevant because the final dataset was obtained after sequential screening, fixation of negligible factors, LHS-based augmentation, and expert-guided domain optimization, so the level distribution of the realized campaign had to be verified after completion of the full experimental sequence. Figure 7 shows that the realized design retained an approximately uniform marginal use of factor levels across the varied inputs. For each varied factor, the level counts sum to 150, i.e., one observation per batch in the full campaign. Balanced marginal counts support data reliability by limiting level-frequency bias and reducing the risk that apparent factor effects, feature-importance rankings, or machine-learning sensitivities are driven by over-represented levels. They also improve interpretability because comparisons among factor levels are based on broadly comparable empirical support, which helps distinguish controlled process effects from residual batch-to-batch variability.

As designed, the curing classes CC_1_ (Day 1) and CC_28_ (Days 2–28) are binary, while the remaining variables are treated as five-level factors. For the curing-temperature variables CT_1_ and CT_28_, the numeric settings behind Levels 1 and 2 coincide by construction (both 20 ^°^C), which preserves orthogonality with respect to the categorical curing class while providing replicates at the reference temperature.

All fresh UHPC tests (temperature, slump flow, V-funnel efflux time, and air content) were conducted in accordance with DIN EN 12350-1/ -7/ -8/ -9, using apparatus that met or exceeded the accuracy requirements specified in the respective standards (for example, the thermometer used for fresh concrete temperature measurement had an accuracy of ±0.5 ^°^C compared to the ±1 ^°^C tolerance required by the standard). Mechanical tests followed DIN EN 196-1: for each batch, fresh UHPC was cast into 40 × 40 × 160 mm prisms, flexural strength was determined in three-point bending, and compressive strength was measured on the resulting 40 × 40 × 80 mm halves. Reported strengths are the means of three prisms (flexural) and six half-prisms (compressive) per batch, ensuring that each data point is underpinned by multiple replicate measurements. All experiments were performed in the same laboratory, using the same mixer, tooling, and personnel under a controlled ambient temperature of 20 ^°^C, thereby constraining environmental variability and supporting the reproducibility of the measurements.

For quality-control annotation and construction of the curated modeling set, the 150 experimental records were evaluated using a defined two-step procedure. The *integrity check* denotes a completeness check of the mechanical target variables required for supervised analysis, in particular CS_28_ and FS_28_, and identifies batches for which critical target values are absent. The *plausibility screen* denotes a domain-informed consistency assessment in which fresh-UHPC properties and strength measurements are compared with the empirical distribution of the campaign and with physically expected UHPC behavior, including incompatible combinations of measurements such as very high slump flow together with prolonged V-funnel efflux time. The *clean range* of a variable denotes the minimum-to-maximum interval spanned by the retained, internally consistent observations after this screening; these ranges are reported in Table 3 and are used as dataset-specific empirical reference intervals, not as general UHPC acceptance limits. Eleven experiments were excluded from the curated modeling set (Table 4) because of missing targets, values that were implausible given the recorded process settings, or documented execution errors. The public archive retains all 150 experiments and flags these anomalous cases for transparency, whereas the curated set used for modeling contains 139 experiments.

**Table 3:** Descriptive statistics for all input factors and output variables in the curated UHPC dataset (139 experiments).

Group	Factor	Symb.	Unit	Mean	Median	SD	Min.	Max.
**Material quality**	Graphite	GRP	kg	0.08	0.09	0.07	0.00	0.22
	Ingredient moisture	IM	kg	3.13	3.15	0.16	2.92	3.36
	Ingredient/Water temperature	IT	^°^C	24.20	25.00	9.05	10.00	40.00
**Particle size distributions** & **measurement errors**	Sand type I	SAI	kg	5.98	6.00	0.59	5.10	6.90
	Sand type II	SAII	kg	10.53	10.50	1.04	8.92	12.07
	Filler type I	FLI	kg	6.00	6.00	0.59	5.10	6.90
	Filler type II	FLII	kg	0.75	0.75	0.07	0.63	0.86
	Superplasticizer	SPP	kg	0.32	0.32	0.02	0.29	0.35
**Mixing condition**	Average power consumption	APW	kW	1.04	1.06	0.19	0.36	1.40
**Fresh concrete properties**	Fresh concrete temperature	FCT	^°^C	26.77	27.00	3.43	17.60	33.30
	Electrical conductivity	EC	V	4.615	4.611	0.029	4.541	4.745
	Air content	AC	%	1.6	1.5	0.8	0.4	7.0
	Slump flow	SF	mm	335.91	340.00	26.35	215.00	395.00
	Funnel runtime	FR	s	7.53	7.00	2.82	4.00	24.10
**Curing conditions**	Curing temperature, day 1	CT_1_	^°^C	24.60	20.00	9.72	10.00	40.00
	Curing class, day 1	CC_1_	Class	—	—	—	1	2
	Curing temperature, days 2–28	CT_28_	^°^C	22.15	20.00	9.38	10.00	40.00
	Curing class, days 2–28	CC_28_	Class	—	—	—	1	2
**Outputs**	Compressive strength, day 28	CS_28_	MPa	109.83	110.57	11.83	85.06	135.71
	Flexural strength, day 28	FS_28_	MPa	16.98	17.29	3.60	8.15	24.57

Variables are grouped by factor class (material quality, particle-size distributions and potential dosing errors, mixing energy consumption, fresh UHPC properties, curing conditions, and final mechanical properties). For each variable, the empirical mean, median, standard deviation, and minimum and maximum values are reported; the latter correspond to the dataset-specific clean ranges defined above. In particular, the 28-day compressive strength CS_28_ spans 85.06–135.71 MPa around the 120 MPa reference recipe, illustrating the substantial variability in mechanical performance within the explored factor space.

**Table 4:** Experiments flagged as anomalous and excluded from the curated modeling dataset (11 of 150 experimental records).

Exp.	Observed anomaly (value → clean range) and rationale
5	SF = 190.0 mm → 215–395 mm (abnormally low flow; mixture too stiff). Documented batching error; excluded.
17	All measured variables within clean ranges; excluded due to documented operator error (inadvertent composition/volume deviation).
30	Values within global ranges, but 24 h strengths are unusually low for the recorded process (e.g., CS_1_ = 13.36 MPa and FS_1_ = 2.67 MPa, both near the lower bound); suspected abnormal early-age curing; excluded as non-representative.
36	SF = 120.0 mm → 215–395 mm; CS_28_ = 73.72 MPa → 85.06–135.71 MPa. Non-representative rheology and depressed final strength; excluded.
41	Multiple aberrations: SF = 85.0 mm → 215–395 mm; FR missing; AC missing; CS_1_ = 6.65 MPa → 11.36–98.23 MPa; FS_1_ = 1.20 MPa → 1.98–12.23 MPa; CS_28_ = 6.03 MPa → 85.06–135.71 MPa; FS_28_ = 1.42 MPa → 8.15–24.57 MPa; FCT = 37.30 ^∘^C → 17.60–33.30 ^°^C; APW = 1.493 kW → 0.36–1.40 kW. Severe procedural/testing anomaly; excluded.
47	SF = 420.0 mm → 215–395 mm together with FR = 20.00 s (towards the slow-flow end): contradictory fresh UHPC behavior; excluded.
57	All measured variables within clean ranges; excluded due to documented operator error (inadvertent composition/volume deviation).
99	All measured variables within clean ranges; excluded due to documented operator error (inadvertent composition/volume deviation).
101	All measured variables within clean ranges (CS_28_ = 85.70 MPa, close to the minimum of the clean range); excluded due to documented operator error (inadvertent composition/volume deviation).
128	Critical targets missing: CS_1_, FS_1_, CS_28_, and FS_28_ absent; FR and AC also missing; unusable for supervised learning; excluded.
148	CS_1_ = 95.37 MPa (near the upper bound of the clean range) inconsistent with the recorded process conditions; excluded as non-representative.

For each experiment, the principal anomaly is reported as the observed value together with the corresponding clean range (see Table 3), along with a brief rationale for exclusion.

Descriptive statistics for all input factors and output variables in the curated dataset (139 batches) are summarized in Table 3. Within this curated set, the 28-day compressive strength CS_28_ ranges from 85.06 to 135.71 MPa, bracketing the 120 MPa reference recipe. The dataset therefore encompasses a technically relevant spread in mechanical performance, including batches that would meet and batches that would fall short of the nominal design strength. This variation is consistent with the reproducibility challenges observed in industrial UHPC production and provides a basis for studying how process variability can lead to off-specification products, with potential implications for material waste and embodied CO_2_ emissions.

## Usage Notes

The primary data files (.xlsx / .csv) can be opened with standard spreadsheet software (e.g., Microsoft Excel) or imported into common data-analysis environments such as R or Python (e.g., using readxl / readr in R or pandas.read_excel / pandas.read_csv in Python). Users should consult the accompanying README.txt for definitions of all columns, units, and experimental details prior to analysis.

Blank cells (empty fields) denote missing values; in this dataset, six experiments contain missing entries in either fresh UHPC properties or final strength measurements, which can be addressed via iterative, round-robin regression-based imputation (e.g., MICE or scikit-learn’s IterativeImputer)^29,30^. These should be treated as missing (e.g., NA/NaN) rather than interpreted as zeros or errors. Batch entries flagged as anomalous records are clearly annotated and should be handled with care – analysts often exclude or separately examine such records to prevent undue influence on statistical results.

For machine-learning reuse, the modeling task should first be defined by the prediction target and by the stage of the UHPC production chain at which the prediction is intended. For final-property regression, CS_28_ and FS_28_ are the primary target variables. The predictor matrix can contain all other variables available before the target measurement or a purpose-specific subset. For example, a controllable-factor model can be restricted to material-quality, dosing, ingredient-storage conditions, and curing variables, excluding average mixer power (APW) and fresh-UHPC properties because these are measured process responses. An end-of-mix model can additionally include APW and fresh-UHPC properties, and a post-24 h model can additionally include 24 h mechanical properties. Conversely, APW, fresh-UHPC temperature, electrical conductivity, air content, slump flow, or V-funnel efflux time can be selected as targets when the objective is to model mixing demand or fresh-state behavior; in such cases, only upstream raw-material, dosing, storage, and prescribed mixing variables should be used as predictors.

For model evaluation, leave-one-out cross-validation (LOOCV) is recommended as the primary train-test strategy because the curated modeling set contains a limited number of observations and the data originate from a structured DoE. If feature selection or hyperparameter optimization is performed, a nested cross-validation structure should be used. A percentage-based hold-out split, for example 90% training and 10% testing, can also be applied for sensitivity analysis; however, results should be averaged over repeated random splits because a single split can distort the marginal factor-level balance and target-property distribution created by the DoE. Where multiple records originate from the same mixed batch and differ only in curing conditions, grouped splitting by the original batch can be used when the objective is to evaluate generalization to unseen production batches.

## Data Availability

The datasets generated and analyzed during the current study are available in the DaKS - University of Kassel's research data repository, 10.48662/daks-56.2.

## Code availability

All code used in this contribution is openly available in DaKS, the University of Kassel’s research data repository, at 10.48662/daks-56.2. The companion code archive at this DOI contains an R script implementing the Latin hypercube augmentation of the Taguchi screening design and two Jupyter notebooks for post-processing the augmented designs and reproducing the exploratory analyses reported in this work.
